# Gamma suppression correlates with thalamic stimulation therapeutic response in intractable epilepsy

**DOI:** 10.1002/epi.70340

**Published:** 2026-06-23

**Authors:** Zachary T. Sanger, Xinbing Zhang, Thomas Lisko, Hafsa Farooqi, Steffen Ventz, Sandipan Pati, Thomas R. Henry, David P. Darrow, Michael C. Park, Theoden I. Netoff, Robert A. McGovern

**Affiliations:** ^1^ Department of Biomedical Engineering University of Minnesota Minneapolis Minnesota USA; ^2^ Department of Neurosurgery University of Minnesota Minneapolis Minnesota USA; ^3^ School of Public Health Division of Biostatistics & Health Data Science University of Minnesota Minneapolis Minnesota USA; ^4^ Department of Neurology University of Minnesota Minneapolis Minnesota USA

**Keywords:** ANT‐DBS, gamma, neurmodulation, seizures, slow gamma

## Abstract

**Objective:**

In patients with drug‐resistant epilepsy who undergo anterior nucleus of the thalamus (ANT) deep brain stimulation (DBS), efficacy is assessed months after therapy initiation and clinicians have no guidance when choosing stimulation parameters due to the lack of real‐time biomarkers. Here, we identified acute and chronic suppression of slow gamma local field potential (LFP) oscillations (SGOs) (20–50 Hz) in the ANT as a novel electrophysiological biomarker correlated with therapeutic response.

**Methods:**

Participants enrolled in an ongoing prospective ANT‐DBS parameter optimization trial (*N* = 11) were analyzed retrospectively for the effects of stimulation on ANT LFPs. One‐minute baseline and stimulation ANT LFPs were captured across follow‐up visits using a Medtronic Percept, testing different stimulation settings around the Stimulation of the Anterior Nucleus of the Thalamus in Epilepsy (SANTE) trial clinical setting (145 Hz, 90 μs) in the clinic. Aperiodic component detrended ANT LFP power spectral density responses during stimulation were compared against each baseline responses. Participants kept a seizure diary, where responders were those who achieved greater than a 50% seizure frequency reduction compared to their pre‐DBS seizure frequency.

**Results:**

In the seven participants exhibiting SGOs, six were responders. Progressive suppression (“gamma fade”) of SGOs under chronic stimulation correlated with long‐term seizure reduction in five of six responders. Acute stimulation in‐clinic with multiple settings suppressed SGOs in four of five responders, challenging fixed‐programming paradigms, with only one responder using the clinical gold standard parameters at the last follow‐up visit.

**Significance:**

These findings establish SGO suppression as a potential multiscale biomarker for responder identification, parameter titration, and therapeutic tracking for precise, biomarker‐guided intervention.

**Key points**

- Slow gamma local field potential (LFP) oscillation (SGO) suppression and clinical therapeutic response were observed in six of seven SGO participants.
- One SGO non‐responder maintained unsuppressed SGO, suggesting therapy suppression needed.
- “Gamma fade” or persistent gamma suppression in stimulation off state associated with seizure reduction.
- Bayesian surface LFP response modeled relative suppression under different parameters.
- Most responders maximal suppressive setting diverged from conventional clinical parameters.

## 1 INTRODUCTION

Despite advances in pharmacological and surgical therapies, epilepsy remains refractory in approximately one‐third of patients,^1^, ^2^, ^3^ many of whom are ineligible for or have failed resective surgery.^4^, ^5^ This population, affected disproportionately by uncontrolled seizures, heightened risk of sudden unexpected death in epilepsy (SUDEP), and substantial psychosocial burden, represents a persistent and growing global challenge. Neuromodulation therapies, such as vagus nerve stimulation^6^, ^7^, ^8^ (VNS), responsive neurostimulation (RNS),^9^, ^10^, ^11^, ^12^, ^13^ and deep brain stimulation (DBS),^14^, ^15^, ^16^ offer a promising therapeutic alternative for individuals with drug‐resistant epilepsy. However, their clinical deployment remains fundamentally constrained by a critical limitation: unlike movement disorders, where the effects of stimulation are immediately observable (e.g., tremor suppression in Parkinson's disease), the therapeutic response to anterior nucleus of the thalamus (ANT) DBS unfolds gradually over weeks to months, without an accessible biomarker to guide stimulation optimization within the clinic or over time. As a result, stimulation titration is effectively blinded, relying on delayed seizure outcomes and subjective reports, which prolong exposure to uncontrolled seizures, a period of elevated mortality and morbidity, while delaying therapeutic benefit.

This temporal disconnect introduces significant clinical uncertainty. Stimulation parameters are typically adjusted empirically, drawing on fixed protocols from pivotal trials,^14^ tolerability thresholds, or retrospective seizure diaries. This approach delays treatment refinement and prolongs exposure to ineffective therapy. The absence of a physiologic signal linking acute stimulation to long‐term seizure outcomes has hindered progress in three essential domains^1^: rapid, data‐driven stimulation titration^2^; early identification of treatment responders; and^3^ development of closed‐loop neuromodulation strategies tailored to individual network states. Although surrogate metrics such as impedance changes,^17^ autonomic fluctuations,^18^, ^19^, ^20^ and spectral power dynamics^21^, ^22^, ^23^, ^24^, ^25^, ^26^, ^27^, ^28^, ^29^ have shown potential for seizure detection and/or forecasting, none has provided a reliable or targetable marker of stimulation efficacy.

Here, we bridge this translational gap by identifying a local field potential (LFP) signal, slow gamma oscillations (SGOs) (20–50 Hz), in the ANT, which manifests as a periodic peak near beta oscillations. This oscillatory activity was observed during routine clinical programming, where acute suppression in the clinic and chronic attenuation (“gamma fade”) across visits was associated with long‐term seizure reduction. These findings establish SGOs as a putative biomarker of ANT‐DBS efficacy, providing a rational, physiology‐based framework for stimulation therapy optimization. By enabling immediate and long‐term feedback on therapeutic engagement, this biomarker has the potential to transform epilepsy neuromodulation from a delayed, empirically guided process into a precision therapy informed by target engagement biomarkers.

## 2 MATERIALS AND METHODS

### 2.1 In clinic visit

All participant data (*N* = 11) shown have been collected as part of an ongoing University of Minnesota clinical trial (#NCT05493722) investigating the efficacy of personalized DBS stimulation parameters to reduce the broadband power within the ANT. Within this trial, a large LFP dataset collected from many refractory epilepsy participants has provided the opportunity to retrospectively analyze any biomarkers that may be present and how DBS neuromodulation may impact potential biomarkers. The analysis shown here is exploratory and not the original intent of the trial; however, this retrospective analysis may aid in future clinical trials to specifically investigate this analysis.

Participants have all received Medtronic Percept Implantable Pulse Generators (IPG) for its ability to simultaneously stimulate and record. Some participants were implanted with Medtronic DBS Legacy leads (*N* = 2, 3389) and others received the latest Medtronic Sensight DBS leads (*N* = 9, B33005). All participants underwent personalized lead targeting, where leads were placed trans‐ventricularly near the mamillothalamic tracts^30^ with Contacts 1 and 2 programmed as monopolar stimulating contacts and 0–3 capturing the differential sensing configuration.

The LFP data shown here are composed of immediate baseline recordings and stimulation recordings of nine different settings tested around the Stimulation of the Anterior Nucleus of the Thalamus in Epilepsy (SANTE) trial^14^ clinical setting (145 Hz, 90 μs). The 9 setting 3 × 3 setting grid is limited by the Medtronic Percept BrainSense artifact reducing stimulation parameter options: 125, 145, or 165 Hz and 60, 90, or 120 μs pulse width. Stimulation amplitude was titrated clinically accordingly by the physician across follow‐up visits. Electrode impedance measurements were taken at each follow‐up and participant seizure diary was documented by the research team.

For each setting, 1 min of baseline activity was recorded prior to each setting with 1 min of stimulation. Participants were awake, attentive, and interacting with the staff throughout data collection. Stimulation amplitude, frequency, and pulse width were adjusted across different settings while preserving the total electrical energy delivered (TEED). During in‐clinic recordings, stimulation cycling and soft start were disabled because the participants could tolerate this change and it reduced potential artifacts observable in the LFP recordings.

TEED=I^2*f*pw/impedance

Acute effects of stimulation could not be studied in participants with Legacy leads due to artifacts that caused broadband shifts in the noise floor. For these participants, results in Figures 2, 3, 4 show changes only with stimulation off.

### 2.2 LFP pre‐processing and signal analysis

For improved neural oscillation visualization and comparison of stimulation parameters, we developed a pre‐processing and analysis pipeline based on pre‐existing tools in the neuromodulation field. Epilepsy participants' neural activity in the time domain, as compared to other neural conditions, can manifest differently depending on the individual's seizure activity landscape. In addition, Medtronic Legacy leads were not designed to be utilized for neural recording and it has been shown that these leads can be susceptible to fluid ingress.^31^, ^32^, ^33^ We have observed electrocardiography (ECG) artifacts in participants with ANT DBS Sensight leads.^34^ So, when QRS cardiac complexes were visually perceivable in the raw LFP recordings, unilateral or bilateral cardiac artifacts were removed using a template subtraction method in the time domain.

The PSD of the time domain LFP data was then estimated using a short‐time Fourier transform (STFT) frequency transform approach using multiple orthogonal windowing functions, often referred to as the multitaper transform.^34^ This approach provided lower PSD variance while maintaining frequency main lobe resolution at the cost of the phase data. Low‐frequency artifact such as stimulation on/off switching transients, ictal discharges, or movement artifact can be difficult to characterize, so these artifacts were removed in the frequency domain by rejecting windows across the STFT response before averaging across times to determine the LFP recording PSD.

Once the filtered PSD was generated for each stimulation trial with an associated prior baseline, the PSD was detrended into the periodic component using a least squares regression fit approach with the Fitting Oscillations and One Over F fitting (FOOOF)^35^ logarithmic function to estimate the *1/f* aperiodic component.^34^

$$1/f\;\left(\mathrm{dB}/\surd \mathrm{Hz}\right)=-1*\log_{10}\left(k+f^{x}\right)+b$$

Because the aperiodic component is believed to be the background activity of that brain region, the aperiodic fit of the baseline recording was used to detrend both the baseline and stimulation responses for each setting in the event stimulation had an effect on the 1/*f* shape.

### 2.3 Statistical analysis and Gaussian process regression estimation

To assess the association between being a responder and having an SGO baseline response, a Fisher exact test was used comparing responders/non‐responders and SGO/no SGO participants in the population.

To compare the effect of ANT‐DBS on SGOs, the mean SGO response within participants during baseline and stimulation was compared across visits using a single‐tail Wilcoxon signed‐rank test.

Due to sparse sampling, the seizure reduction correlation with mean SGO power suppression was calculated using a gaussian process regression model of the seizure reduction to obtain a seizure response over time. Some participants tracked their seizure frequency more frequently than they visited their neurologist. Using this model with both seizure reduction and mean SGO power z‐scored, if the data were correlated, the model should effectively predict mean SGO power at a specific time during follow‐up. The statistical significance is determined by the 95th percentile of the surrogates (*N* = 200), where we compared the seizure reduction's model log likelihood of predicting the SGO mean power across visits compared to the time shuffled surrogates of the SGO power.

An LFP response surface of the participant's frequency and pulse width parameter space was estimated using a gaussian process regression fit of the ANT LFP response to nine initial stimulation parameter configurations. Given the heterogeneous peak morphology and varying peak width, for each participant, we defined the patient specific SGO frequency span as the bounded area under the curve where the stimulation and baseline spectral responses overlapped at the 1/f aperiodic components. Additional information on the peak variability and morphology in the study cohort is shown in Figure S2. A gaussian process regression was generated using a matern 3/2 kernel with a length scale of 2.5 and sigma of 0.1. Over time, SGOs were modulated by stimulation resulting in a decreased signal to modulate with stimulation. To better visualize the surface of mean relative differences between stimulation parameters across visits that modulate SGOs, later visits where SGO had already been suppressed (±0.025 dB stimulation parameter relative difference) were omitted. These visits were still included in the statistical analysis. To demonstrate differences across settings, the response surface was normalized by the mean response of the nine measured settings. If a stimulation parameter other than 145 Hz, 90 μs exhibited a greater reduction in SGOs, that research optimized setting was tested by the participant at home.

## 3 RESULTS

### 3.1 Patient disposition

Between 2021 and 2024, a total of 14 adults (≥18 years) with medically intractable focal epilepsy and clinically indicated ANT‐DBS implants were prospectively enrolled in an ongoing institutional review board (IRB)–approved clinical trial at the University of Minnesota evaluating stimulation parameter optimization. Of the 14 enrolled participants, 11 were included and 3 excluded from analysis due to insufficient in‐clinic data and lack of seizure frequency tracking. All 11 participants had bilateral ANT‐DBS leads implanted and were enrolled with Medtronic sensing enabled Percept systems.

**FIGURE 1:**
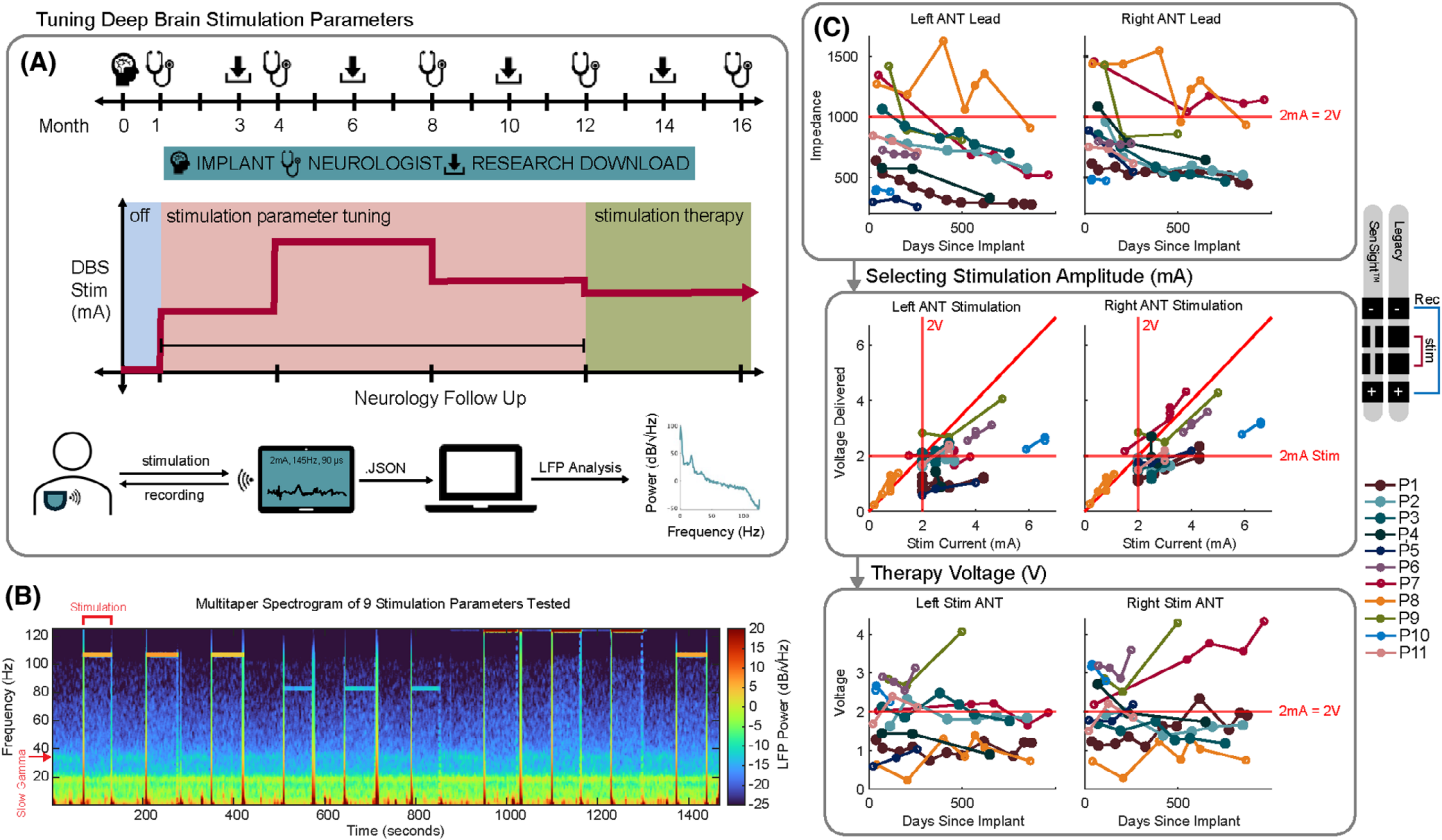
Participant visits and ANT‐DBS therapy tuning. (A) Intracranial signals were recorded at each clinical visit following implantation of their Percept PC device. Neurologist visits typically occur 1 month after implantation and then repeated every 3–4 months thereafter. Data are downloaded from the Percept device at each of these visits storing up to 60 days of neurological signals stored at 10 min intervals. (B) Spectrogram of neural signals recorded at 250 Hz in‐clinic while applying different stimulation parameters. A higher LFP power is represented with a warmer color. Note the peaks around 105, 85, and 125 Hz occur during stimulation at those frequencies (or aliased from stimulation frequencies above Nyquist), whereas lower frequencies indicate power from neural sources. (C) Left and right ANT‐DBS lead Contacts 1 and 2 impedance across in‐clinic follow‐up visits. The conversion between milliamps (mA) and volts is represented as a red line when assuming a 1 kΩ electrode impedance. The trade off given the lead impedance between delivered voltage and stimulation current is shown in the second row of figures. Finally, the delivered stimulation voltage across visits is shown for both hemispheres in all participants. ANT, anterior nucleus of the thalamus; DBS, deep brain stimulation; LFP, local field potential.

Stimulation was enabled at the first neurologist follow‐up visit, as shown in Figure 1A, using clinically standard settings based on the SANTE pivotal trial (145 Hz, 90 μs).^14^ After enrollment, the participant's stimulation amplitude was then titrated by the neurologist over follow‐up visits based on the seizure frequency feedback from the participant and side‐effect profile. Participant demographics, seizure onset, seizure types, devices, and clinical treatment plan are shown in Table 1.

**TABLE 1:** Participant population.

	Slow gamma	Responder (R)/non‐Responder (NR)	Stimulation Parameters	Lead Impedance (Ω) and voltage (V)	Lead	Days in study	Anti‐seizure medications (dose)	Additional devices	Relevant history/imaging findings/prior procedures	Sex/age	Seizure onset	Seizure types/symptoms
1	Yes	R	4.3 mA, 125 Hz, 50 μs, none	*L*: 287 *V* = 1.23 *R*: 513 *V* = 2.21	Sensight	773	Diazepam (5 mg, 1/day), valproate (500 mg, 3/day), oxcarbazepine (300 mg, 2/day), Phenytoin (100 mg, 4/day)	No	Traumatic brain injury; Primarily left frontal encephalomalacia, Smaller areas of right temporal and occipital Encephalomalacia	M 30s	Left frontal/temporal	Focal impaired consciousness Focal impaired consciousness → tonic–clonic
2	Yes	R	3.2 mA, 165 Hz 60 μs, 1 M/4 M	*L*: 653 *V* = 2.09 *R*: 556 *V* = 1.78	Sensight	683	Lacosamide (100 mg, 2/day), carbamazepine (100 mg, 10/day)	No	Perinatal ICH; bilateral parietooccipital encephalomalacia	F 20s	Bilateral frontal Bilateral posterior quadrant	Focal preserved consciousness Focal impaired consciousness
3	Yes	R	2.5 mA, 145 Hz, 90 μs, 1 M/5 M	*L*: 704 *V* = 1.76 *R*: 471 *V* = 1.18	Sensight	753	Esclicarbazepine acetate (800 mg, 2/day), clobazam (10 mg, 2/day), felbatol (600 mg, 3/day)	VNS (off)	Left anterior temporal lobectomy, topectomy	F 40s	Left temporal/left posterior temporal/parietal	Focal preserved consciousness → Focal impaired consciousness Generalized tonic–clonic
4	Yes	R	2.7 mA, 125 Hz, 90 μs, 1 M/5 M	*L*: 332 *V* = 0.90 *R*: 643 *V* = 1.74	Sensight™	647	Cenobamate (600 mg, 1/day), clonazepam (1.5 mg, 1/day), diazepam (5 mg prn), lacosamide (200 mg, 2/day)	VNS (on)	None	M 50s	Bilateral Frontal/temporal Frontal polar	Focal impaired consciousness Focal impaired consciousness with observable manifestations: hyperkinetic behavior Focal impaired consciousness → generalized tonic–clonic
5	Yes	R	4.5 mA, 165 Hz, 120 μs, 1 M/5 M	*L*: 243 *V* = 1.09 *R*: 568 *V* = 2.56	Sensight™	392	Lacosamide (200 mg, 3/day), valproate (500 mg, 2/day)	No	None	M 30s	Left temporal Right frontal/temporal	Focal Impaired consciousness
6	Yes	R	5.3 mA, 165 Hz, 60 μs, 1 M/4 M	*L*: 679 *V* = 3.60 *R*: 780 *V* = 4.13	Legacy	248	Cenobamate (150, 200 mg 1/day), felbamate (400 mg, 8/day), levetiracetam (4× 500 mg XR, 500 mg bt), phenytoin (100 mg, 3/day), topiramate (25, 50 mg, 2/day)	RNS (on)	Parietooccipital encephalomalacia	F 30s	Left frontal/temporal Left parietal/posterior quadrant	Focal preserved consciousness Focal impaired consciousness Focal impaired consciousness → Generalized tonic clonic
7	Yes	NR	3.6 mA, 165 Hz, 90 μs, 1 M/3 M	*L*: 520 *V* = 1.87 *R*: 1140 *V* = 4.1	Legacy	961	Oxcarbazepine (300 mg, 2/day), cenobamate (200 mg, 4/day, 100 mg, 1/day)	No	None	M 30s	Bilateral frontal	Focal preserved consciousness Focal preserved consciousness → Focal impaired consciousness
8	No	NR	1.5 mA, 145 Hz, 60 μs, 1 M/5 M	*L*: 909 *V* = 1.36 *R*: 934 *V* = 1.40	Sensight™	918	Lamotrigine (150 mg, 2/day), levetiracetam (500 mg, 2/day), zonisamide (7/day)	No	T2 Hyperintense right cerebellar hemisphere lesion	M 30s	Bilateral frontal	Focal impaired consciousness Generalized tonic–clonic
9	No	NR	4.0 mA, 145 Hz, 90 μs, 1 M/5 M	*L*: 813 *V* = 3.25 *R*: 859 *V* = 3.44	Sensight™	868	Carbamazepine (300 mg, 8/day), levetiracetam (500 mg, 8/day), lamotrigine (150 mg, 4/day), perampanel (12 mg, 1/day 2 mg, 1/day)	VNS (off)	Left inferior temporal gyrus topectomy Left Inferior Parietal/occipital topectomy	M 50s	Left posterior Hemisphere Left frontal/temporal	Focal preserved consciousness Focal impaired consciousness with observable manifestations: distal and proximal automatisms Generalized tonic–clonic
10	No	NR	4.8 mA, 125 Hz, 90 μs, 1 M/5 M	*L*: 400 *V* = 1.92 *R*: 480 *V* = 2.30	Legacy	429	Lamotrigine XR (150, 50, 200 mg, 1/day), oxcarbazepine (600 mg, 3/day), zonisamide (300, 100, 300 mg, 1/day), clonazepam (0.5, 0.5, 1 mg, 1/day)	Pacemaker (on)	Right temporal lobectomy	F 40s	Left temporal/parietal Right posterior quadrant	Focal preserved consciousness with observable manifestations: aphasia Focal impaired consciousness with observable manifestations: automatisms
11	No	NR	3.7 mA, 145 Hz, 60 μs, 1 M/5 M	*L*: 697 *V* = 2.58 *R*: 630 *V* = 2.33	Sensight™	366	Carbamazepine (300, 300, 200 mg, 1/day), clonazepam (1 mg, 1/day), lamotrigine (200 mg, 3/day), levetiracetam (500 mg, 6/day)	No	None	F 40s	Right temporal	Focal preserved consciousness Focal preserved consciousness → Focal impaired consciousness Focal preserved consciousness → Focal impaired consciousness → Generalized tonic–clonic

At each follow‐up visit, participants underwent a standardized stimulation parameter test set of nine settings (125, 145, 165 Hz, 60, 90, 120 μs) centered around the clinical standard SANTE trial stimulation parameter of 145 Hz, 90 μs, as shown in Figure 1A,B. To estimate the changes in participants' broadband LFP response to different stimulation parameters, a gaussian process regression fit was used to estimate a response surface drawing from the mean LFP response under the initially tested settings. Using the response surface, further testing was conducted around the stimulation settings that resulted in the greatest suppression of neural activity for each participant.

Impedance in each ANT hemisphere (lead Ring 1 and 2 to allow for sensing 0–3) was recorded and plotted in Figure 1C. We observed that the majority of participants with ANT lead implants exhibited impedances lower than 1 kΩ, with many implants around 500 Ω. Although electrical stimulation therapies utilize constant current stimulation for the safety benefits, therapy efficacy may be dependent on the delivered electrical field (volts/meter) and its distribution through the local neural tissue as opposed to the current amplitude defined.^36^ This trade off in each participant between the current programmed and delivered voltage is shown based on the impedance measurement extracted by the Percept device as shown in Figure 1C. As a result, many participants were programmed with amplitudes ranging from 4 to 6 mA to then receive ≈2–3 volts of delivered electrical stimulation.

### 3.2 Local field potentials and seizure frequency

**FIGURE 2:**
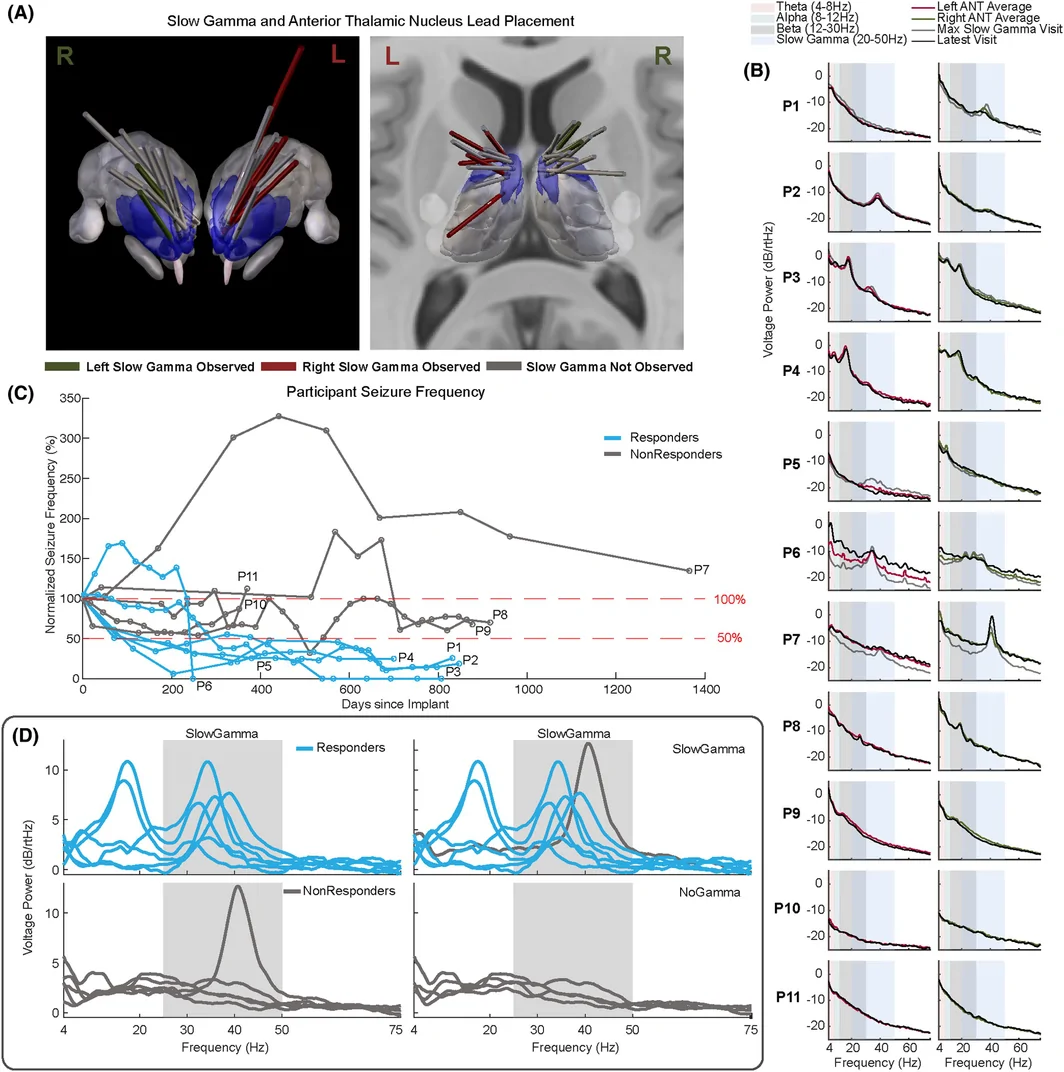
Observed neural activity in responders and non‐responders to ANT‐DBS. (A) Coronal and axial views of all participants' bilateral lead placements plotted using a standardized atlas with LeadDBS. Red (left) and green (right) leads demonstrate where SGOs were observed. Blue shaded tissue represents the ANT. (B) PSD responses without stimulation across all participants with the neural Berger bands are shown for biomarker identification. For each participant, the average and latest PSD is shown with an additional max SGO PSD in participants where SGOs were observed. (C) Seizure reduction normalized by the pre‐DBS monthly seizure frequency for all participants. Blue represents responders and gray represents non‐responders. (D) Participants are grouped by their max 1/*f* baseline SGO detrended PSDs based on responder/non‐responder or SGO/no SGO categorization. The colors represent each participant's responder status based on 2C. ANT, anterior nucleus of the thalamus; DBS, deep brain stimulation; PSD, power spectral density; SGO, slow gamma oscillation.

While testing different stimulation parameters across follow‐up visits, we recorded bilateral ANT LFPs during stimulation on and off conditions. LFP activity was sensed in each participant through the 0 and 3 ring contacts of the lead. Lead placement is shown in Figure 2A within a standardized Morel atlas using LeadDBS. The leads were then colored red (left) or green (right) if an SGO (20–50 Hz) peak was observed in the LFP multitaper power spectral density (PSD) estimate.

Across the 11 participants, we observed a SGO peak in seven participants (P1–P7) in the baseline PSD response with stimulation off as shown in Figure 2B. This SGO peak was predominantly in one hemisphere, not consistently on a particular side, with varying peak frequencies. The left and right ANT PSD is shown with the max SGO visit, average PSD across visits, and most recent visit PSD for each participant. To better understand the utility of this SGO response during stimulation, participant seizure frequency was recorded through seizure diaries at every research visit and is shown in Figure 2C. Participants were deemed a responder to ANT‐DBS if they achieved a seizure reduction greater than 50% by their last follow‐up visit. To reduce reporting variability and seizure relapsing, a three‐sample moving average was used to extract seizure frequency from the raw reported data. Some non‐responders had a short period of 50% reduction; however, they quickly relapsed back to their pre‐DBS seizure frequency.

A Fisher exact test was used to understand the association between responders and those with an observable baseline SGO peak. There was a statistically significant association between SGOs and responder status (*p* = 0.015).

Participants were sorted into groups based on responder status and whether SGOs were observed as shown in Figure 2D. Of the seven participants with observable SGOs, six were responders. Notably, the only non‐responder in this group was the one participant in whom we could not modulate SGO activity with DBS. All of the participants without SGOs were non‐responders.

### 3.3 DBS modulation of ANT slow gamma oscillations (SGOs)

**FIGURE 3:**
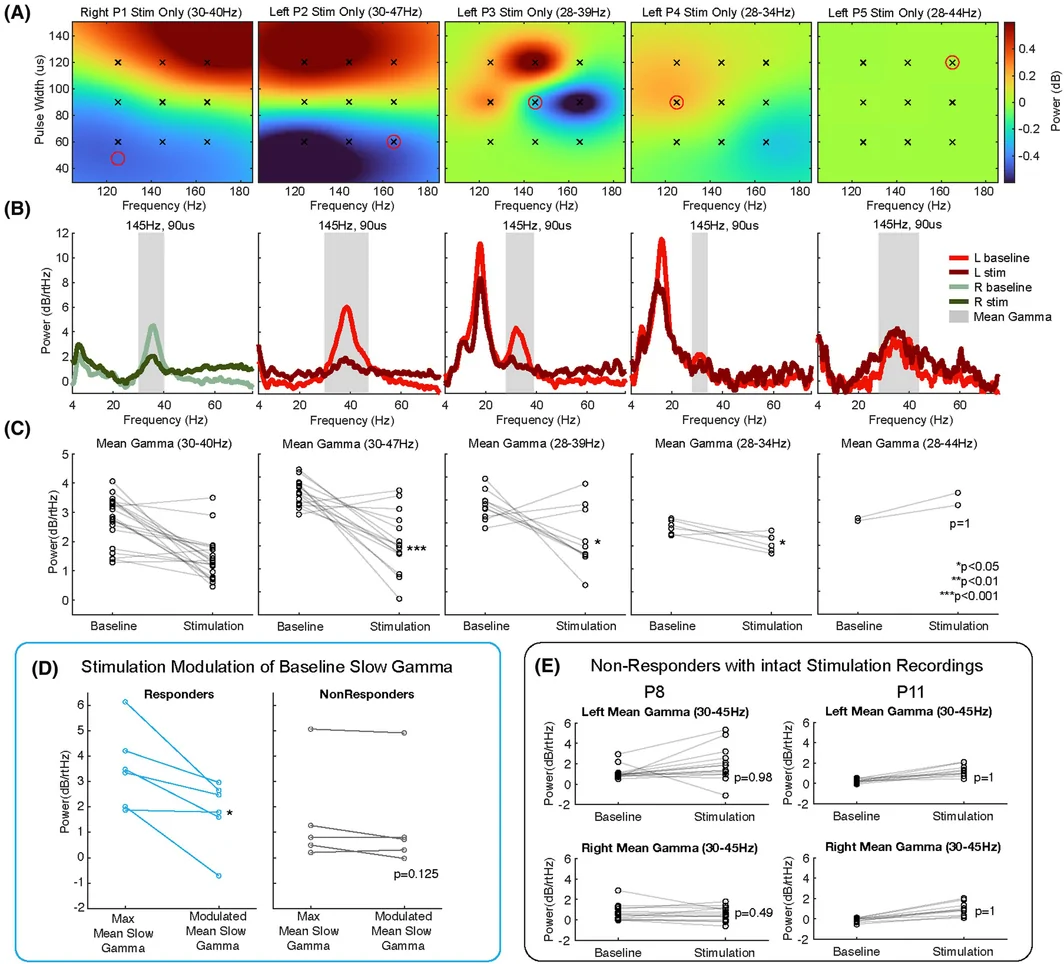
Slow gamma modulation in the clinic. (A) In all responder participants without stimulation recording artifacts, average gaussian process regression surfaces across visits for each participant where SGOs were observed are shown. Each SGO frequency range is shown in the title and also shown as a shaded region in 3B. The black x's represent the stimulation parameter test set and the red circle indicates their latest setting. Cool colors demonstrate a larger reduction in SGOs as compared to the other stimulation parameters. (B) Average baseline and stimulation 1/*f* detrended PSD under 145 Hz, 90 μs across participants in 3A. (C) Mean SGO response in each baseline and stimulation test pair across visits with the associated Wilcoxon signed‐rank one‐sided *p*‐value for SGO suppression during stimulation. (D) Modulation of SGOs shown across all participants organized by responders and non‐responders. A Wilcoxon signed‐rank comparison between the max observed SGO and most recently recorded SGO LFP response in both groups to show change in SGO across visits under stimulation. (E) Bilateral SGO changes in two non‐responders during ANT‐DBS under the clinical setting. Non‐significant *p*‐values determined through a Wilcoxon signed‐rank test. LFP, local field potential; SGO, slow gamma oscillation.

Although observation of an SGO signal in the ANT may be a baseline signal seen in responders, we were interested in the ability of ANT‐DBS to modulate this neural signal. In Figure 3A, the mean SGO LFP PSD response is shown across the frequency vs pulse‐width response surface. Individualized research‐optimized stimulation parameters based on the SGO LFP are shown on each surface as a red circle. Participants 1–3 (P1–P3) demonstrated larger baseline SGOs and SGO suppression, leading to higher dB differences between stimulation parameters.

The standard ANT‐DBS clinical setting, 145 Hz, 90 μs, is shown in Figure 3B across these participants to demonstrate the average PSD response observed across all visits during standard clinical stimulation. Figure 3C demonstrates the change between baseline and stimulation for each stimulation setting test across all the visits shown in Figure 3B. A Wilcoxon signed‐rank test demonstrated a single‐tail statistically significant reduction in SGOs during stimulation (P1: *p* = 5.8E‐5, P2: *p* = 3.4E‐5, P3: *p* = 7E‐3, P4: *p* = 4E‐3, P5: *p* = 1). The P6 and P7 stimulation modulation could not be determined due to multiple artifacts throughout all stimulation recordings. All P6 and P7 PSDs during stimulation had a roughly 5 dB greater background noise level than during baseline, resulting in the noise floor completely covering an observed SGO signal during baseline. In addition, low beta suppression was observed in Participants 3(R), 4(R), and 8(NR) and is shown in supplemental information where no superior relative LFP suppression response was observed across stimulation parameters in the responders.

The chronic effects of stimulation on SGOs in all participants, not just participants where stimulation recordings were not corrupted, are shown in Figure 3D. Statistically significant suppression of SGOs was observed in responders when comparing the visit with max mean SGOs compared to their current mean SGOs. Finally, in participants with non‐corrupted stimulation LFP responses who were not responders, no acute statistically significant SGO suppression was observed, as shown in Figure 3E.

### 3.4 “Gamma fade”: ANT DBS suppression of SGOs over time correlates with seizure reduction

**FIGURE 4:**
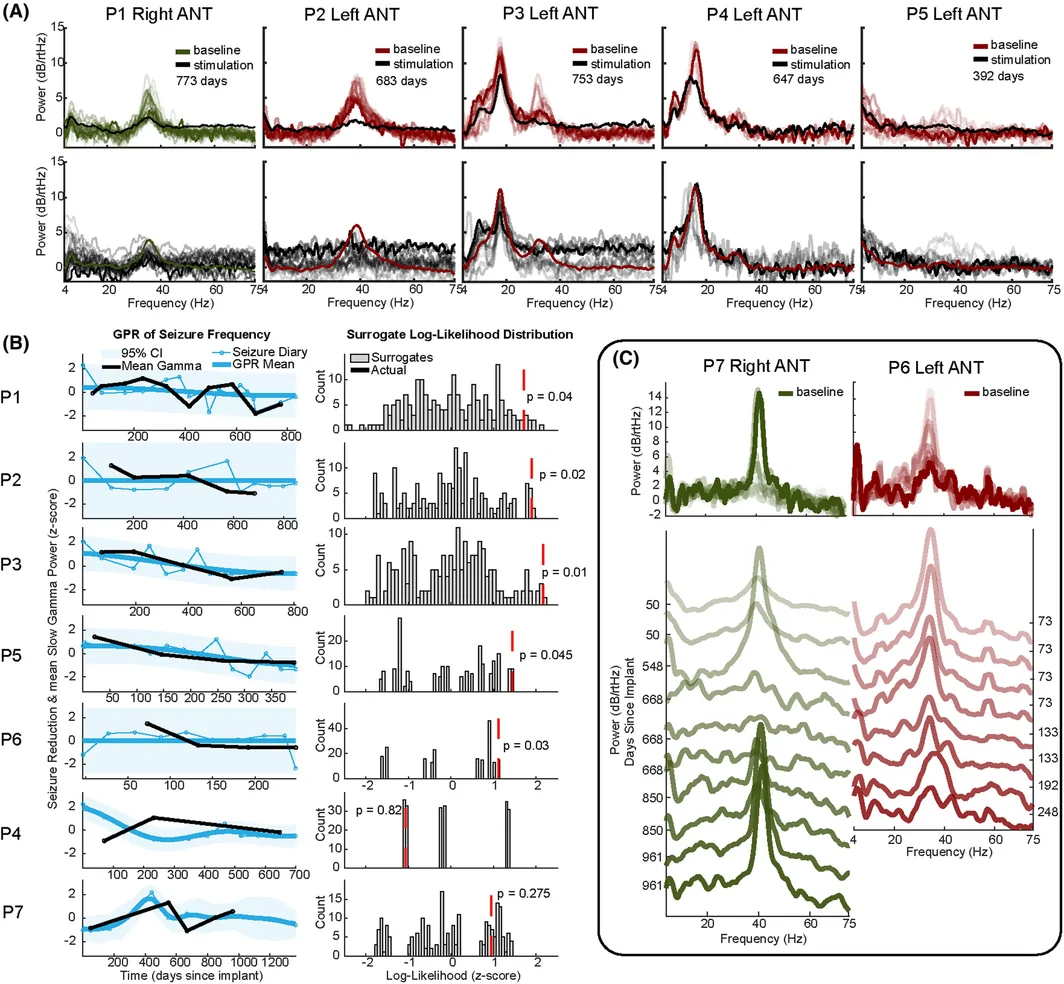
Baseline gamma fade across visits. (A) Responder participants with Sensight leads and an SGO peak with the change in SGOs during stimulation and baseline across visits. A more transparent detrended PSD (right [green] and left [red]) represents an earlier clinical visit. Baseline is shown with an average stimulation PSD over visits (top) or stimulation is shown with an average baseline PSD (bottom). In Participants 2 and 3 (P2 and P3), a rise in the noise floor is observed at higher frequencies. (B) Seizure reduction normalized by the pre‐DBS seizure frequency correlated with five of seven SGO participants is shown. Seizure diary reported seizure reduction is shown in blue correlated in time with the observed mean visit baseline SGO power in black. For each participant, a histogram of the log likelihood of predicting the mean SGO power from the seizure reduction model is shown. The log likelihood of slow‐gamma time shuffled surrogates are compared to the log likelihood of the actual data (shown as a red dotted line). (C) Legacy lead participants, one responder (P6) and one non‐responder (P7), baseline detrended PSD across visits. Stimulation is not shown due to artifact corruption during stimulation. DBS, deep brain stimulation; PSD, power spectral density; SGO, slow gamma oscillation.

Not only can SGO reduction be observed acutely within the clinic, but a steady reduction in baseline SGOs was observed across visits. During in‐clinic follow‐up, temporarily disabling stimulation allowed for observation of baseline SGO activity changes over time under chronic ANT‐DBS therapy. We have named this reduction “gamma fade,” as the impact of stimulation over multiple visits suppressed the SGO activity observed over time. Across visits within responders, baseline SGO power slowly decreased and SGOs observed during stimulation were consistently suppressed as shown in Figure 4A.

To investigate the connection between the baseline SGO fade and the patient's improvement with DBS, we assessed the seizure reduction response's ability to predict the mean SGO amplitude in the clinic across visits. Five of six responders showed statistically significant correlation of seizure frequency with mean SGOs (Figure 4B). Four of five non‐responders demonstrated no SGO peaks and therefore this relationship could not be assessed. The one non‐responder who did have an SGO peak (P7, Figure 2B,C,E) that was unable to be modulated with stimulation showed no significant relationship between seizure frequency and mean SGOs.

Finally, P6 and P7 are participants with Legacy leads and exhibited multiple artifacts during stimulation, rendering acute stimulation effects unobservable. With stimulation off, their baseline activity can be observed (Figure 4C) over multiple visits. P7 is the only participant in our dataset that exhibited gamma that has worsened alongside a fluctuating seizure frequency and more severe seizures. Of note, stimulation did not appear to affect SGO power amplitude throughout their course. P6 has demonstrated consistent SGO reduction since receiving their Percept system, allowing us to record in‐clinic baseline LFP signals.

## 4 DISCUSSION

Our discovery of ANT SGOs as a biomarker of DBS efficacy has the potential to advance therapeutic paradigms for drug‐resistant epilepsy by resolving a fundamental disconnect: the inability to assess stimulation effects in real time. We demonstrate that SGOs operate across three clinically actionable timescales: (1) as a potential predictor prior to implant, given that 85.7% of participants with SGOs following implant were responders; (2) a dose‐titration signal in the clinic, where acute SGO suppression led to positive responses; and (3) a longitudinal feedback metric where consistent SGO suppression (gamma fade) correlated with effective therapy. This multiscale utility addresses the critical limitation of current practice, where clinicians optimize DBS blindly while patients remain at elevated risk of seizures during months of titration.

The strong association between baseline SGOs and response (*p* = 0.015) suggests that thalamic SGOs may identify patients with ANT‐connected epileptogenic networks. Notably, the biomarker's value lies not merely in detection but in modifiability. The non‐responder with unmodifiable increasing SGOs over time (P7) underscores that this oscillation may be pathologic and must be disrupted to provide therapeutic benefit. This aligns with emerging DBS principles where biomarker responsiveness, not just presence, determines clinical utility. Although SGOs were suppressed with the clinical and multiple research parameters in the clinic, chronic suppression of SGOs was demonstrated under patient‐specific parameters in five of six responders, challenging the fixed‐parameter approach of historical trials. This also lays the foundation for establishing patient‐specific stimulation paradigms for future trials.

Although our findings demonstrate a correlation between participants' SGO peak modulation by ANT‐DBS and response rate, the origin of this slow‐gamma oscillation could not be identified in this study because recordings were limited to the ANT. Furthermore, it is not known how it correlates with seizure severity, seizure types, seizure onset, or the mechanism by which it fades over time. The “gamma fade” phenomenon (progressive baseline reduction correlating with seizure improvement) suggests that ANT‐DBS induces enduring network reorganization. No prior studies have examined this phenomenon in humans, but one pilot study in a single ANT‐DBS responder (81% seizure reduction) demonstrated a strong slow‐gamma peak and fade over time, consistent with our findings.^37^ Although not specifically noted in the article, this does independently illustrate these results. Further studies to understand the laterality of SGOs could better explain why we predominantly observed unilateral SGOs.

In addition, alpha/low beta suppression was observed in three participants and occurred simultaneously with slow gamma suppression in two of three participants. Alpha and low beta have been shown to be elevated during seizure events,^38^ although given that our participants were awake and alert in the clinic, these spectral alpha/low beta signals were not captured during a tonic–clonic seizure, where it is noted that 16 ± 2 Hz activity is elevated during an event. Chronic simultaneous capture of both low beta and slow gamma at home may better explain the connection between these alpha/low beta signals and slow gamma signals as they pertain to epileptic states, events, and the network pathology.

The ANT is well connected within the hippocampal circuit of Papez with bidirectional connections to the amygdala and mesial frontal cortical structures, and receives inputs through the mammillothalamic tracts, thalamocortical radiations, and the fornix.^39^, ^40^ Functionally, the ANT is believed to facilitate communication through theta synchrony between mesial temporal and neocortical structures.^40^, ^41^, ^42^, ^43^ Although it is a possibility that this SGO occurs locally, current ANT‐DBS mechanisms hypothesize that ANT‐DBS inhibits incoming pathological activity and restores normal physiological outputs from the ANT.^44^, ^45^ These SGOs may not originate in the ANT, but arrive through input connections or are generated through reciprocal connections with another area.

Slow gamma rhythms are seen in the hippocampus and arise through connections between the CA1, CA3, and dentate gyrus.^46^, ^47^, ^48^, ^49^ Given that the hippocampus connects to the ANT through the fornix and mammillothalamic tracts, this could be one possible origin of the slow‐gamma oscillations in ANT. Further supporting this, one study has shown that anterior dorsal placement of ANT‐DBS leads resulted in outcomes with the highest seizure reduction^30^ and that this area receives inputs from the mammillothalamic tracts.^50^ Proper placement has been verified post‐implantation in ANT‐DBS participants by utilizing hippocampal evoked potentials during ANT stimulation, demonstrating that target placement contains strong connections from the hippocampus.^51^ In addition, case studies have shown that patients with strong hippocampal evoked potentials during ANT‐DBS have been correlated with good seizure control.^52^ Combined, these results support the hypothesis that SGOs originate within the circuit of Papez and can lead to successful ANT‐DBS outcomes. If these oscillations arrive from the hippocampus, then hippocampal decoupling observed in responders with other neuromodulatory therapies may explain the improvement we observe.

However, the ANT is bidirectionally connected to the neocortex through thalamocortical radiations and connections with the cingulate cortex.^53^ The cingulate cortex, a region with bidirectional connections to the ANT within the circuit of Papez, has been shown to be well connected to the frontal, temporal, and parietal cortices.^54^ A pediatric epilepsy study found a correlation between frontotemporal lobe epilepsy with impaired executive function and damaged, less developed thalamocortical pathways.^55^ Another study in frontal lobe epilepsy participants found that after 3 years, 14 of 16 participants who underwent anterior thalamic radiation disconnection as part of their frontal lobe resections were seizure‐free, whereas only 11 of 31 were seizure‐free in patients who had those tracts preserved.^56^ During working memory tasks, SGOs have also been observed in the medial prefrontal cortex coupled to theta oscillations within the hippocampus.^57^ Therefore, not only do thalamocortical radiations play a role in frontotemporal lobe epilepsy, but cortical structures associated with executive function tasks may generate SGOs.

SGO observation raises the provocative question of whether chronic SGO suppression disrupts pathologic thalamocortical synchrony. The correlation between fade kinetics and seizure reduction timing hints at plastic changes underlying therapeutic effects, although causal studies are needed. Mechanistically, SGOs may reflect aberrant thalamic gating of cortico‐limbic traffic; its suppression could normalize hyperexcitable circuits.

Translational opportunities are immediate: sensing‐enabled devices (e.g., Percept) already capture this signal, enabling prospective trials comparing SGO‐guided vs empiric titrated stimulation parameters. Future work should investigate whether pre‐implantation network mapping (e.g., tractography) and/or seizure network localization through stereo‐electroencephalography (sEEG) can predict “gamma positive” phenotypes, optimizing patient selection for ANT‐DBS. Pre‐implantation SGO signal localization may also foster investigation into new stimulation targets interconnected with the ANT.

There were limitations to this study, and findings should be interpreted accordingly. First, although the relationship between SGO suppression and seizure reduction was robust across participants, causal inference is limited by the observational design. Whether the suppression of SGOs is necessary and sufficient for therapeutic effect remains to be tested in a large sample size. Second, although our findings are promising, the sample size is modest, and larger multicenter studies will be necessary to validate the generalizability of SGOs as a biomarker. Within our cohort, SGO peak center frequency and peak morphology were varying, warranting further studies to characterize a standard morphology to identify prospectively. Third, In our modest sample size cohort, we observed varied, atrophied anterior thalamic nuclei and observed within‐subject relative suppression, as opposed to absolute suppression. Better understanding these dynamics in diverse anatomy across a large patient cohort would further validate our findings. Finally, the clinical utility of this biomarker in real‐time closed‐loop paradigms has yet to be explored. In one participant, we observed that SGOs were maximally observable during the day in a participant with primarily nocturnal seizures.^58^ Others have demonstrated the positive effects on ictal activity through stimulation during interictal states.^59^ Further investigation of SGO suppression adaptively during interictal states could preserve efficacy while reducing side effects like ANT‐DBS sleep disruption.^60^

This study was also limited to the capabilities of the Percept platform. The Percept platform, due to filtering and sampling rate, practically allows for observing LFP neural activity between 5 and 100 Hz. In addition, due to the sense‐friendly stimulation parameters, stimulation frequencies were limited to higher frequencies, excluding the practical study of lower frequency epilepsy therapy paradigms.^61^ Second, Medtronic Percept lead electrodes cannot be programmed as both a stimulating and sensing contact, preventing the exact spatial interface for both stimulating and sensing. Third, artifact contamination during stimulation limited analysis in two participants, emphasizing the need for improved artifact mitigation strategies in chronic sensing systems. Artifact contamination in discontinued, Legacy leads will diminish with next‐generation directional electrodes and artifact‐suppression algorithms.

## 5 CONCLUSION

This work establishes the potential of SGOs as both a biomarker and a fundamental axis of network pathophysiology, bridging acute ANT‐DBS effects to chronic seizure control. The convergence of adaptive stimulation with SGO tracking could aid in new refractory epilepsy neuromodulation therapy management, where therapy adapts not only to disease states but to individual network dynamics in real time. Crucially, this approach shifts the paradigm options away from only reactive seizure suppression to proactive circuit modulation, where tuning stimulation to electrophysiological states of therapeutic readiness rather than clinical events. SGOs thus provide the first evidence‐based roadmap for closed‐loop DBS in epilepsy, offering a measurable substrate to accelerate optimization, personalize parameters, and ultimately mitigate the prolonged mortality risk inherent to traditional trial‐and‐error programming.

## AUTHOR CONTRIBUTIONS

Z.T.S., T.I.N., and R.A.M. conceptualized the study. R.A.M. and T.I.N. served as the principal investigators and supervised the research protocol. X.Z., Z.T.S., H.F., and T.L. coordinated patient recruitment and data collection. Z.T.S., X.Z., H.F., T.I.N., S.V., and R.A.M. contributed to data validation, imaging analysis, and statistical analysis. Z.T.S. drafted the initial manuscript. T.R.H., S.P., D.P.D., and M.C.P. acted as physician collaborators on the research. All authors reviewed and approved the final version of the manuscript.

## FUNDING INFORMATION

This work has been supported by the National Institute of Neurological Disorders and Stroke (U01NS124616, TIN, RAM). Zachary Sanger is a 2024–2025 MnDrive Brain Conditions Fellow and his time conducting research reported in this publication was supported by the University of Minnesota’s MnDRIVE (Minnesota’s Discovery, Research and Innovation Economy) initiative.

## CONFLICT OF INTEREST STATEMENT

The authors declare no conflicts of interest. Z.T.S. completed this research work as part of his PhD dissertation research. This work was completed prior to accepting full time employment at Medtronic. Z.T.S. held a part‐time 1‐year graduate internship role within Medtronic during this dissertation work; that research work was unrelated to the findings published here.

## ETHICS STATEMENT

This study was reviewed and approved by the University of Minnesota’s Institutional Review Board and research was conducted in accordance with the Declaration of Helsinki. We confirm that we have read the Journal’s position on issues involved in ethical publication and affirm that this report is consistent with those guidelines.

## CONSENT

Written informed consent was obtained from all patients for their study participation and publication of this study.

## Supporting information


Figure S1
Figure S2

## Data Availability

The data that support the findings of this study are available from the corresponding author upon reasonable request.
