# Supplementary material for: Gamma suppression correlates with thalamic stimulation therapeutic response in intractable epilepsy
Source: Epilepsia. 2026 Jun 23;67(9):4828–42. doi: 10.1002/epi.70340 (PMC13440434; doi:10.1002/epi.70340)
Supplement: Supplementary file 1 [file EPI-67-4828-s001.docx]

**Supplemental Figures:**

**Supplemental Figure 1: ANT DBS LFP Modulation**

A) Linear regression of responder participants 1, 2, and 3 demonstrated large SGO reduction across multiple visits to show the relationship between baseline SGOs and the amount of SGO reduction observed. B) In participants 8 (NR), 3 (R), and 4 (R), statistically significant beta suppression is shown. Relative beta suppression across the 9 tested settings is shown with the 145Hz, 90us baseline and stimulation PSD shown with associated Wilcoxon rank sum p-values.

**Supplemental Figure 2: SGO vs Non-SGO Peak Properties**

A) Local peaks with the maximum peak prominence detected within the SGO frequency range, 20-50Hz. The annotation shows the peak frequency and peak prominence. B) Boxplot of SGO peak prominence grouped by No-/SGO participants. All participants labeled as SGO have peak prominence greater than 1dB, while non-SGO participants have peak prominence less than 1dB.
